# Divergent selection on, but no genetic conflict over, female and male timing and rate of reproduction in a human population

**DOI:** 10.1098/rspb.2013.2002

**Published:** 2013-12-07

**Authors:** Elisabeth Bolund, Sandra Bouwhuis, Jenni E. Pettay, Virpi Lummaa

**Affiliations:** 1 Department of Animal and Plant Sciences, University of Sheffield, Sheffield S10 2TN, UK; 2 Edward Grey Institute, University of Oxford, Oxford OX1 3PS, UK; 3 Institute of Avian Research, An der Vogelwarte 21, 26386 Wilhelmshaven, Germany; 4 Department of Biology, University of Turku, Turku 20014, Finland

**Keywords:** sexually antagonistic selection, genetic correlation, heritability, life history

## Abstract

The sexes often have different phenotypic optima for important life-history traits, and because of a largely shared genome this can lead to a conflict over trait expression. In mammals, the obligate costs of reproduction are higher for females, making reproductive timing and rate especially liable to conflict between the sexes. While studies from wild vertebrates support such sexual conflict, it remains unexplored in humans. We used a pedigreed human population from preindustrial Finland to estimate sexual conflict over age at first and last reproduction, reproductive lifespan and reproductive rate. We found that the phenotypic selection gradients differed between the sexes. We next established significant heritabilities in both sexes for all traits. All traits, except reproductive rate, showed strongly positive intersexual genetic correlations and were strongly genetically correlated with fitness in both sexes. Moreover, the genetic correlations with fitness were almost identical in men and women. For reproductive rate, the intersexual correlation and the correlation with fitness were weaker but again similar between the sexes. Thus, in this population, an apparent sexual conflict at the phenotypic level did not reflect an underlying genetic conflict over the studied reproductive traits. These findings emphasize the need for incorporating genetic perspectives into studies of human life-history evolution.

## 1. Introduction

The timing and rate of reproduction are important fitness determinants in many species and the sexes often differ in their phenotypic optima regarding when and how often to reproduce [1]. This leads to divergent selection pressures between the sexes, or sexually antagonistic selection. However, an evolutionary response to selection requires genetic transmission to following generations because only the heritable component of a trait is passed on to offspring [2]. Furthermore, most traits are expressed by the same genes in both sexes (i.e. the same loci encode the trait in males and females) leading to an often strong positive genetic correlation between the trait in males and females. This intersexual genetic correlation constrains the ability of the sexes to respond independently to selection and can lead to one sex displacing the other from its phenotypic optimum (so-called intralocus sexual conflict [3,4]). This conflict can potentially be resolved by sex-specific gene expression and sexual dimorphism. However, because these processes often need to evolve over long time scales and their evolution can be hampered by other processes that prevent the evolution of complete dimorphism, we can often expect to encounter unresolved sexual conflict in nature [5–7]. Indeed, evidence suggests that such conflict is widespread (reviewed by Bonduriansky & Chenoweth [8]) and it has become a dominating theme in evolutionary studies on non-human animals in recent years [4].

To demonstrate sexually antagonistic selection, one needs to show: (i) that the phenotypic selection pressures on the trait differ between the sexes, (ii) that the trait has significant genetic variation (i.e. there must be additive genetic variance) and shows a strong positive intersexual genetic correlation (i.e. a shared genetic architecture), so that the ability of the sexes to evolve independently is constrained, and (iii) that the genetic covariance between the trait and relative fitness is positive in one sex but negative in the other. To study this, a general framework that has been developed to study selection and evolutionary response to selection can be applied (reviewed in [3]). Recently, several theoretical and empirical papers [9–12] have advocated the use of the Robertson–Price identity (sometimes called the ‘secondary theorem of natural selection’ [3,13,14]) in studies of evolutionary change. The Robertson–Price identity states that the expected change in mean phenotype between generations is equal to the additive genetic covariance between the trait and relative fitness (or the extent to which heritable genetic differences among individuals determine both the trait and fitness). Thus, if we observe in a population that the highest fitness is achieved in males by individuals that reproduce late in life but in females by individuals that reproduce early in life, the Robertson–Price identity allows us to predict sex-specific evolutionary responses over one generation to this observed phenotypic directional selection. Importantly, the focus on the genetic covariance means that the estimate is unbiased by the covariance between the non-heritable environmental component of the trait and fitness (e.g. if individuals that grow up under abundant resource availability reproduce earlier [15,16]).

Studies of sexual conflict tend to focus on polygamous species, where there are large differences between the sexes in the variance of reproductive success. However, males and females might experience different phenotypic selection on a number of life-history traits even under monogamy [17]. For example, a study on a serially monogamous human population from preindustrial Finland found that improved mating success led to an increase in reproductive success and that this relationship was stronger in men than in women [18].

In human studies, sexual conflict has recently been the subject of increasing interest [19]. For example, the optimal family size has been recognized to be influenced by the sex-specific cost of reproduction and the availability of alternative reproductive options owing to divorce and extra-pair fertilizations [20]. Further, the cost of reproduction was found to be higher in females than in males in humans [21]. However, these studies focus on the phenotypic level, while an evolutionary response to selection only occurs if the trait has a genetic basis and is genetically correlated with fitness. Despite this continued focus on the phenotypic level, an evolutionary perspective is gaining momentum and results showing that humans are still subject to natural selection and are still experiencing evolutionary change are rapidly accumulating (reviewed in Stearns *et al*. [22]). A number of studies have demonstrated significant heritability of traits related to reproduction (reviewed in [22]) and other studies have demonstrated a genetic correlation between reproductive traits and fitness [23–25]. Thus, we might expect an evolutionary response. However, these studies have been limited by focusing only on females, or by using a twin design or parent–offspring regression, which largely precludes the estimation of intersexual genetic correlations [3]. Instead, pedigree data that span several generations allow the use of all levels of relatedness to disentangle genetic from environmental effects and also allow the estimation of intersexual genetic correlations. Pedigree data have been used very extensively in studies of non-human animals (reviewed in [26,27]), but have only recently been applied in studies on humans, mainly owing to the extreme scarcity of such data. Nevertheless, the few available studies on humans clearly demonstrate the usefulness of a pedigree approach [24,25,28,29]). For example, Pettay *et al*. [28] found a significant heritability of age at last reproduction in females but not males in a Finnish sample. Another recent study used the ongoing Framingham Heart Study to provide first evidence that evolution of a contemporary American population may be constrained by genetic conflict over medically important traits [29]. In this population, selection favoured shorter women and taller men, but female height was negatively genetically correlated with cholesterol in men, potentially leading to maintenance of overall higher cholesterol levels despite selection for reduced cholesterol in females. Overall however, it remains largely unknown whether heritabilities and genetic correlations with fitness are different between the two sexes in humans (see [28–30] for exceptions).

We here apply quantitative genetic methods that are well tested in the field of evolutionary biology to a large longitudinal dataset from Finland on humans. This dataset contains accurate records of births, marriages and deaths in seven preindustrial populations and allows us to build pedigrees with up to 12 generations of data depth. It is thus ideally suited to establish whether sexually antagonistic selection occurred on the genetic level, in this case on traits related to reproductive timing and rate during a period of natural reproductive conditions (i.e. high infant mortality and prior to the use of contraceptives). The detailed records allow us to statistically control for a range of environmental factors (such as aspects of culture) that are known to influence life-history traits in humans, for example socioeconomic status and patterns of wealth inheritance [31,32].

## 2. Material and methods

### (a) Data selection

We use demographic data (of 69 691 individuals) from seven farming and fishing human populations (‘parishes’) in Finland. Starting in the eighteenth century, the Lutheran church was obliged by law to accurately record all births, marriages and deaths in every parish [33]. Our data come from three inland parishes (Ikaalinen, Pulkkila and Tyrvää), two coastal parishes (Hiittinen and Kustavi) and two inland eastern parishes, located in current Russia (Jaakkima and Rautu). Because divorce was forbidden, remarriage permitted only if individuals were widowed, and extramarital affairs were punishable [34], extra-pair paternity rates are likely to have been substantially lower than the current median worldwide extra-pair paternity rate of 9%, and probably ranging between 1.7 and 3.3% as also suggested for modern populations with high paternity confidence [35]. Such low levels of extra-pair paternity are insufficient to bias quantitative genetic estimates qualitatively [36].

We *a priori* decided to restrict our analyses to individuals with complete records of their life history until the age of 45 for women and 50 for men to focus on individuals whose potential reproductive period has been fully documented. Thus, we excluded individuals who either died before or were not tracked until adulthood at age 15. We also excluded individuals surviving to adulthood and with complete life histories, but who never reproduced because the timing and rate of reproduction can only be recorded in individuals who do reproduce. Post hoc inspection revealed that in the current full dataset, 99% of women but 93% of men who ever reproduced had stopped reproducing by the age of 45 and 50, respectively. The more stringent criterion of 99% for men would require men to have complete records until the age of 59 inclusive. All (except one male) who fulfilled all other subsetting criteria (see below) indeed had complete records until this age. Individuals who died before these ages were included if they fulfilled all other criteria because running all analyses on a subset of the data that included only individuals that survived to the age of 50 years did not significantly influence any of the results. To ensure complete records of the potential reproductive period of all offspring, we also required that all offspring of each included individual had complete records of their life history until the age of 45 for women and 50 for men. In addition, we required complete information for a number of parameters that were entered as fixed and random effects in the statistical models. Because social class was related to key life-history traits in these populations [31], we used the occupations of men to assign a two-level socioeconomic status to him and his wife: landowning versus landless [32]. We also accounted for twinning status (singleton or twin [37]), firstborn son (heir; yes or no) to control for inheritance of status from parents [38], parish (seven different parishes), maternal identity (to account for the non-independence of individuals born in the same family) and birth cohort divided into 20-year intervals [39]. This resulted in a subset of 1229 females and 1093 males, born between 1621 and 1937. For quantitative genetic analyses, the full pedigree of 69 691 individuals was pruned to include informative individuals only (i.e. 5787 individuals, including 2194 mothers and 2152 fathers with a maximum pedigree depth of 12 generations).

We used four measures to capture the variation in timing and rate of reproduction; age at first reproduction, age at last reproduction, reproductive lifespan (calculated as age at first—age at last reproduction) and reproductive rate (calculated as number of offspring/reproductive lifespan). Because the calculation of a rate requires two values that are separated in time, we included only individuals with more than one offspring, and excluded individuals with two offspring if those were twins, resulting in a subset of 903 females and 859 males for reproductive rate. To assess the degree of sexual dimorphism on the phenotypic level in these traits and in the number of grandchildren born, we used univariate ANOVAS with sex as the main fixed effect and the reproductive trait as the response variable (table 1). Note that because the study includes only reproductive individuals from each sex, unequal average number of offspring and grandoffspring for males and females is possible.

**Table 1.** Sexual dimorphism in traits related to timing and rate of reproduction. (Means ± s.d. are provided along with *F-* and *p*-values from univariate ANOVAS comparing the traits in the two sexes.)

trait	females	males	*F*_1,2320_	*p*
age at first reproduction	26.5 ± 5.3	28.7 ± 6.1	91.2	<0.0001
age at last reproduction	34.4 ± 6.9	37.4 ± 8.0	95.5	<0.0001
reproductive lifespan	7.9 ± 7.0	8.7 ± 7.6	6.4	0.012
grandchildren	7.7 ± 9.5	8.4 ± 9.7	3.19	0.074

	females	males	*F*_1,1760_	*p*
reproductive rate	0.52 ± 0.3	0.54 ± 0.3	0.98	0.32

Thirteen per cent of men and 10% of women had more than one spouse over their lifetime (range 1–3, averaging 1.06 ± 0.39 in females and 1.14 ± 0.38 in males (average ± s.d.). The number of offspring born over a lifetime ranged from 1 to 17 with an average (±s.d.) of 3.7 ± 2.7 for females that ever reproduced and 4.0 ± 2.8 for males that ever reproduced. Child mortality was high with only 65 ± 36% (average ± s.d.) of offspring surviving to adulthood (15 years). Fitness was quantified as the number of grandchildren born, to control for any quantity versus quality trade-off at the offspring stage [32], and to include grandparental effects [40,41] and ranged from 0 to 62 (with 337 females and 255 males having 0 grandchildren and with an average ± s.d. of 7.7 ± 9.5 for females that ever reproduced and 8.4 ± 9.7 for males that ever reproduced, number of grandchildren born thus conformed to a Poisson distribution.). While number of grandchildren born is arguably the biologically most relevant fitness measure in a human population [22], it includes attributes of the offspring when calculating parental fitness, which is sometimes regarded as problematic [42,43]. We therefore validated our results by using lifetime reproductive success (number of children born, LRS) as our fitness measure. Requiring full information only on the offspring, rather than the grandoffspring level also allowed us to use an extended dataset comprising 5311 women and 5392 men. This did not qualitatively change the results and we thus present results from the grandoffspring models only.

### (b) Phenotypic selection gradients

We performed phenotypic selection analyses and quantitative genetic analyses in the R-package *MCMCglmm* [44], which uses an iterative Bayesian approach suitable for non-Gaussian data. *MCMCglmm* provides meaningful error estimates for derived variables by direct sampling from the posterior distribution. To test for sexually antagonistic selection on traits related to timing and rate of reproduction, we first investigated the relationship between each of the focal four traits and fitness (grandchildren) in models that included the interaction between sex and both the linear and the quadratic term for each trait. Models included socioeconomic status, twin status, firstborn son status and parish as fixed effects and birth cohort and maternal identity as random effects. Reproductive trait values were standardized within sexes prior to the analysis (mean of zero; s.d. of one) and we used grandchildren as a Poisson response variable. The 95% Bayesian credibility intervals for the interaction term indicate whether the linear and quadratic slopes of the relationship between trait and fitness differed significantly between the two sexes. To further elucidate sex-specific phenotypic selection patterns and to facilitate comparison with previous studies, we then calculated standardized phenotypic selection gradients separately in the two sexes. Following Lande & Arnold [45], we used standardized reproductive trait values and relative grandchildren (the grandchildren of an individual/the mean number of grandchildren of individuals of that sex in the dataset). Both reproductive lifespan and reproductive rate are variables that are derived from age at first and age at last reproduction. To avoid problems with multicolinearity and to maximize statistical power [46], we therefore chose to use univariate rather than multivariate models. Importantly, a multivariate analysis assumes that all the traits and environmental factors that could cause the focal trait to covary with fitness have been identified and included in the model [9,45]. This assumption is very difficult to fulfil in studies of natural populations [9]. We thus performed a Bayesian implementation of univariate linear mixed-effect models, with the standardized reproductive trait as a linear and a quadratic predictor and relative grandchildren as a Gaussian response variable. Thus, the linear coefficient represents the univariate standardized phenotypic linear selection gradient [3] and the doubled (following [47]) quadratic coefficient can be interpreted as stabilizing (negative sign) or disruptive (positive sign) selection gradients [45]. All phenotypic selection models were run with a prior with *V* = 1 and a degree of belief parameter (nu) of 0.002.

### (c) Quantitative genetic analyses

We employed a Bayesian version of a linear mixed-effects model approach (the ‘animal model’) which has gained popularity in recent decades in evolutionary biology [26,27,48]. It provides a powerful means to use all the information available in complex, natural pedigrees to estimate additive genetic variances and covariances. The use of all levels of relatives (e.g. siblings, grandparents and aunts/uncles) confers substantial benefits over twin and sibling designs. The latter, which are often used in human studies, can be genetically similar owing not only to additive genetic effects but also owing to non-additive effects such as dominance and epistasis. This is not the case for less closely related individuals [49]. Further, unlike twin analyses, the animal model allows the estimation of intersexual genetic correlations as well as the separation of genetic effects from cultural inheritance, at least to an appreciable degree. Hence, it is being successfully applied in a growing number of human studies [24,25,29]. The animal model also allows us to control for specific confounding factors by adding them as fixed or random effects in the model.

To allow comparison with the phenotypic analyses, we used relative grandchildren as the response and modelled all traits using Gaussian distributions. From the posterior distribution of the genetic variances and covariances, we calculate heritabilities as *V*_A_/*V*_P_. We also calculate maternal effects and genetic correlations along with their 95% Bayesian credibility intervals.

By coding a trait as two separate traits in males and females (treating it as a sex-limited trait), it is possible to run a bivariate model and extract sex-specific heritabilities and the intersexual genetic correlation. To obtain sex-specific genetic correlations between traits and fitness, we used three-trait models with the trait in males, the trait in females and relative grandchildren (fitness, measured on an individual basis) as the three response variables. Results remained qualitatively the same when relative grandchildren was treated as a sex-limited trait and coded as two separate traits in the two sexes, resulting in models with four responses (both reproductive trait and fitness sex-separate). The genetic correlations between reproductive traits and fitness were estimated in four separate models, one for each trait. All models included socioeconomic status, twin status, firstborn son status and parish as fixed effects and birth cohort and maternal identity as random effects. The reported heritabilities are therefore estimated after removing the variation due to the fixed effects. To minimize autocorrelation among samples, models were run for 1 000 000 iterations (after an initial burn-in of 10 000 iterations), and every 1000th iteration sampled for a total of 1000 samples from the posterior distribution. We specified a weakly informative prior by partitioning the phenotypic variance of the traits of interest evenly among each random effect (and covariances to zero) and specifying a degree of belief parameter (nu) equal to the size of the matrix (3 for a trivariate model). Estimates were robust to varying the degree of genetic control specified in the prior (0.95 versus 0.05) and to an alternative prior specifying a *V* of 1 and a nu of 2.002 (for a trivariate model). To account for the effects of the genetic correlations between traits (see the electronic supplemental material, table S1), we attempted to run a multivariate analysis with all four reproductive traits (separately in the two sexes) and fitness in one single model. However, this model failed to reach convergence and we thus present results from the trait-specific models described above.

We used R v. 2.15.0 [50] for all statistical analyses. Data is available from the Dryad Digital Repository: http://doi.org/10.5061/dryad.4h071.

## 3. Results

### (a) Sexual dimorphism in traits related to reproductive timing and rate

Three of the four reproductive traits showed significant sexual dimorphism (table 1). Women started reproducing 2 years earlier than men, and ended reproduction 3 years earlier. This resulted in a reproductive lifespan that was on average 1 year shorter in women than in men. There was a non-significant trend for males to have a higher number of grandchildren. By contrast, both sexes reproduced at similar rates, with on an average of one child born every second year during the reproductive lifespan.

### (b) Phenotypic selection

**Figure 1.**
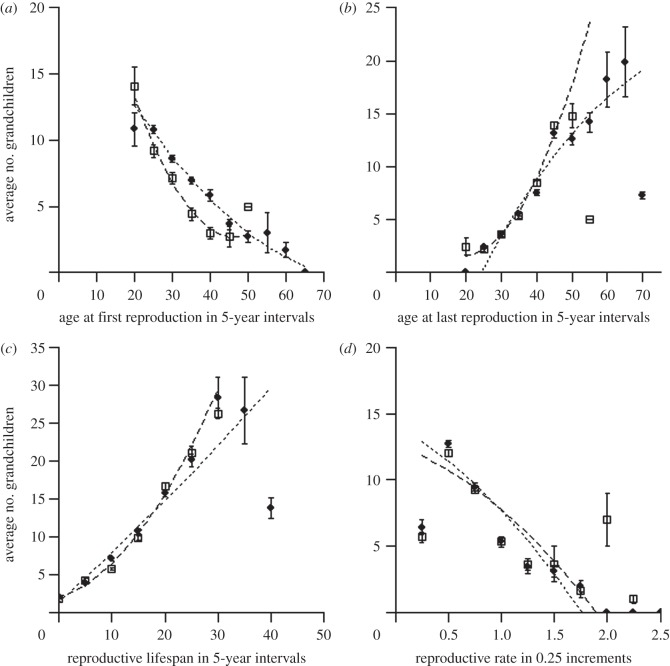
Sex-specific correlations relating the average (±s.e.) number of grandchildren produced (fitness) to (*a*) age at first reproduction, (*b*) age at last reproduction, (*c*) reproductive lifespan and (*d*) reproductive rate. The raw data are grouped here for visual purposes only, whereas analyses (see main text) are performed on ungrouped data. Lines represent the best fit from second-order polynomial regressions weighted by the sample size of each group of phenotypic mean on age (*a–c*) or rate (*d*). Females are denoted by empty squares and dashed regression lines, males by filled diamonds and dotted regression lines.

Figure 1 provides an overview of the relationship between traits related to reproductive timing and rate and fitness, based on grouped raw data. We estimated the differences between the sexes in the linear and quadratic slopes of the relationship between traits and fitness (table 2). This showed that the linear selection for earlier reproduction was stronger in women. Further, the quadratic relationship between fitness and both age at first reproduction and reproductive lifespan was different in the two sexes (table 2). We further estimated linear and quadratic selection gradients in the sexes separately in a Bayesian implementation of univariate linear mixed-effects models (table 2). In women, there was selection for an earlier onset of reproduction and a lower reproductive rate (table 2 and figure 1*a*,*d*). Further, a later age at last reproduction and an increased reproductive lifespan lead to higher fitness. In addition, a positive quadratic relationship for both traits indicated that intermediate values conferred lower fitness benefits than expected from the linear relationship. In men, selection on all traits was generally linear, with very weak quadratic selection gradients. Thus, men were selected for an earlier age at first and a later age at last reproduction, a longer reproductive lifespan and a slower reproductive rate. The linear selection gradients mean that a woman who advanced her age at first reproduction by one phenotypic standard deviation gained 3.1 grandchildren, while a man gained 2.3 grandchildren. Similarly, an increase in reproductive rate by 1 s.d. resulted in a decrease of 1.4 and 2.0 grandchildren in women and men, respectively. A delay in age at last reproduction by 1 s.d. resulted in a gain of 4.5 and 4.0 grandchildren, respectively. Finally, an extension of the reproductive lifespan with 1 s.d. resulted in a gain of 5.2 grandchildren in women and 6.1 grandchildren in men.

**Table 2.** Sex-specific standardized linear (*β*) and quadratic (*γ*) selection gradients for age at first reproduction (AFR), age at last reproduction (ALR), reproductive lifespan (RL) and reproductive rate (RR), with their associated 95% Bayesian credibility intervals. (The interactions between sex and the linear term (sex : *β*) and sex and the quadratic term (sex : *γ*) were estimated in models including both sexes.)

trait	sex	*β* (CI)	*γ* (CI)	sex : *β* (CI)	sex : *γ* (CI)
AFR	females	−0.39 (−0.47 to −0.32)	0.074 (0.013 to 0.11)	−0.15 (−0.30 to −0.016)	0.025 (−0.058 to 0.11)
	males	−0.27 (−0.35 to −0.18)	0.014 (−0.026 to 0.049)		
ALR	females	0.58 (0.50 to 0.63)	0.14 (0.062 to 0.19)	0.006 (−0.11 to 0.13)	0.12 (0.025 to 0.22)
	males	0.48 (0.41 to 0.54)	−0.037 (−0.078 to 0.0027)		
RL	females	0.67 (0.63 to 0.75)	0.17 (0.12 to 0.23)	−0.028 (−0.12 to 0.095)	0.087 (−0.015 to 0.15)
	males	0.73 (0.65 to 0.78)	−0.032 (−0.067 to 0.017)		
RR	females	−0.18 (−0.30 to −0.057)	−0.030 (−0.068 to 0.012)	0.048 (−0.10 to 0.21)	0.030 (−0.029 to 0.096)
	males	−0.24 (−0.34 to −0.12)	−0.016 (−0.053 to 0.019)

### (c) Quantitative genetic estimates of heritabilities and genetic correlations

**Figure 2.**
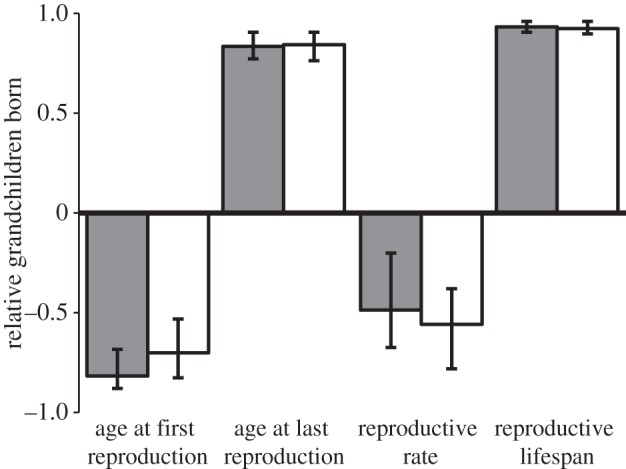
Sex-specific genetic correlations between reproductive traits and fitness, with their associated 95% credibility intervals. Fitness is estimated as the number of grandchildren born relative to the mean number of grandchildren born to individuals of each sex. Grey bars represent females and white bars males.

All reproductive traits were heritable with credibility intervals that did not overlap zero and heritabilities did not differ between men and women (table 3). All traits had a significant maternal effect (table 3). To determine whether the sexes differed in the genetic correlations between traits and fitness, we ran trivariate animal models. The genetic correlations with fitness were generally very strong with the exception of reproductive rate, which was only weakly genetically correlated with fitness. The genetic correlations between trait and fitness were not significantly different, and indeed remarkably similar, between the sexes (table 3 and figure 2). To test whether the genetic correlations between the sexes constrain sex-specific responses to selection, we estimated intersexual genetic correlations. These were generally high, thus potentially constraining the ability of the sexes to respond independently to selection. Reproductive rate, however, tended to be negatively genetically correlated between the sexes (with a wide credibility interval that overlapped zero), indicating the possibility of sex-specific responses to selection (table 3).

**Table 3.** Sex-specific heritabilities (*h*^2^), maternal effects, intersexual genetic correlations (*r*_G F-M_) and genetic correlations between sex-specific traits (age at first reproduction (AFR), age at last reproduction (ALR), reproductive lifespan (RL) and reproductive rate (RR) and fitness (*r*_G trait-fitness_), with their associated 95% credibility intervals. (Fitness is estimated as the number of grandchildren born relative to the mean number of grandchildren born to individuals of each sex.)

trait	sex	*h*^2^ (CI)	maternal (CI)	*r*_G F-M_ (CI)	*r*_G trait-fitness_ (CI)
AFR	females	0.09 (0.037–0.21)	0.09 (0.043–0.21)	0.65 (−0.0007–0.88)	−0.82 (−0.88 to −0.69)
	males	0.19 (0.03–0.38)	0.17 (0.029–0.29)		−0.70 (−0.83 to −0.53)
ALR	females	0.17 (0.078–0.3)	0.07 (0.024–0.15)	0.69 (0.36–0.90)	0.84 (0.78–0.91)
	males	0.12 (0.049–0.23)	0.06 (0.013–0.14)		0.85 (0.77–0.91)
RL	females	0.14 (0.06–0.25)	0.09 (0.029–0.19)	0.75 (0.36–0.88)	0.94 (0.91–0.96)
	males	0.21 (0.086–0.29)	0.13 (0.038–0.24)		0.91 (0.90–0.96)
RR	females	0.07 (0.024–0.24)	0.06 (0.021–0.19)	−0.39 (−0.84–0.49)	−0.49 (−0.68 to −0.20)
	males	0.09 (0.027–0.31)	0.12 (0.024–0.26)		−0.56 (−0.78 to −0.38)
fitness	females	0.12 (0.063–0.19)	0.078 (0.047–0.13)	0.32 (−0.025–0.58)	—
	males	0.14 (0.089–0.27)	0.14 (0.085–0.22)		—

## 4. Discussion

To our knowledge, no previous study on humans fulfils all three requirements needed to demonstrate sexually antagonistic selection on a given trait: a demonstration: (i) that the phenotypic selection pressures on the trait differ between the sexes, (ii) that the trait has significant genetic variation and shows a strong positive intersexual genetic correlation and (iii) that the genetic correlation between trait and fitness is positive in one sex but negative in the other. We found that the phenotypic selection pressures on four traits related to timing and rate of reproduction differed between men and women in a preindustrial Finnish human population. We also found significant heritabilities and generally strong intersexual genetic correlations for these traits, which could potentially lead to sexual conflict. The genetic correlations between trait and fitness were, however, remarkably similar between the sexes, showing that there is no genetic sexual conflict over traits related to the timing and rate of reproduction in this human population. Given persistent selection pressures, we would therefore not predict evolution of increased sexual dimorphism beyond current levels in these traits. Our multigenerational pedigree data allowed us to control for a range of environmental effects that may bias estimates of quantitative genetic parameters, such as inheritance of socioeconomic status and wealth, and therefore allowed us to estimate sex-specific heritabilities and genetic correlations between traits and fitness as well as intersexual genetic correlations with relatively high precision. We are therefore confident that there indeed is no sexual conflict over the traits of interest.

### (a) Phenotypic selection pressures

We found strong linear positive selection for a delayed age at last reproduction in both sexes. However, women ceased to reproduce earlier than men. In preindustrial Finland, previous studies have found that for females, the cessation of reproduction at menopause may be beneficial owing to increasing reproductive conflict over age with own offspring (the ‘Reproductive Conflict hypothesis’ [51]) and because women benefit more in terms of fitness from investing in their grandchildren rather than from continuing to reproduce (the ‘Grandmother hypothesis’ [51–53]). By contrast, no such benefit was found in men [41].

The observed selection for a slower reproductive rate may at first glance seem counterintuitive. However, the reason behind this negative association between reproductive rate and fitness is likely to be that couples responded to high child mortality by increasing their rate of reproduction. In the analysed data, a higher reproductive rate was associated with a lower child survival until the age of 15 (*lmer*: *β*_women_ = −0.18 ± 0.036, *t* = −5.0, *N* = 903, *β*_men_ = −0.15 ± 0.035, *t* = −4.2, *N* = 859). Alternatively, the causality may be reversed, such that high child mortality is a consequence of a high reproductive rate. Either process would result in couples with a slower birth rate and higher child survival having more grandchildren.

### (b) Potential for an evolutionary response

Consistent with previous findings in both humans and wild animals, we found that selection was acting to lengthen the reproductive period, both through selection for an earlier onset and later end of reproduction [23,24,54]. This strong selection, coupled with the high genetic correlation between these traits and fitness, indicates the potential for a strong evolutionary response. This would lead to a lengthening of the reproductive lifespan in both women and men. However, rapid cultural and environmental change makes predictions of evolutionary response difficult in humans, and projections beyond one generation tend to be highly unreliable [22]. In our study, despite strong selection for an earlier start of reproduction and a strong genetic correlation with fitness, age at first reproduction remained relatively high throughout the study period, which covered 350 years (and up to 12 generations). Because the main constraint on marriage was economic [55], we might speculate that the strong genetic correlation between age at first reproduction and fitness was counteracted by a strong cultural pressure to delay reproduction until sufficient economic resources had been accumulated, in the mid- to late 20s in both sexes.

### (c) Quantitative genetic estimates: heritabilities and genetic correlations

Our estimates of the heritabilities and intersexual genetic correlations of traits related to timing and rate of reproduction fall within the range of those reported for other human populations as well as for other vertebrates [1,6,54]. Stearns *et al*. [22] recently reviewed published estimates of *h*^2^ of age at first reproduction and found an across-study average *h*^2^ of 0.11 in five studies performed before 2010. One more recent study estimated a heritability of age at first reproduction between 0.30 and 0.55 [25]. Similar heritabilities have been reported for the age at last reproduction (0.23 for women and 0.34 for men in [56] and 0.42 for women and non-significantly different from zero in men in [28], in a Finnish population sample unrelated to the sample used in this study). Our estimates of the genetic correlations between age at first reproduction and fitness are also consistent with previous findings, as a negative genetic correlation has consistently been found in women [23–25]. Fitness-related traits are theoretically predicted to have a lower heritability and this is generally borne out in empirical studies [49,57]. Our heritability estimates of fitness of 0.12 in females and 0.14 in males are in the range reported for fitness-related traits in humans (*h*^2^ of LRS in women: less than 0.01 and 0.04 in two datasets, respectively, in [25], *h*^2^ of LRS in women: 0.47 in [28], intrinsic rate of increase in women: 0.39 in [23]). Note that while heritability estimates for several reproductive and life-history traits are accumulating for men as well as for women, to our knowledge all previous estimates of the heritability of fitness and of the genetic correlation between age at first reproduction and fitness are based on studies of women only. Thus, very few studies have estimated genetic correlations between the sexes or between traits and fitness. Given the importance of a genetic perspective in studies of evolutionary change [22], this should be addressed by future studies.

A fundamental question in evolutionary biology concerns the maintenance of variation in fitness in wild populations [3]. Bonduriansky & Chenoweth [8] recently reviewed a number of studies that have found a negative intersexual genetic correlation for fitness and suggested that genetic variation in fitness may be maintained to some degree by sexually antagonistic selection. However, data are scarce and more studies are needed from natural populations [8]. In our study, a significant negative correlation would have indicated the presence of sexual conflict in traits that contribute to fitness. However, a near zero or even positive genetic correlation, as was found, indicates that there is unlikely to be strong genetic conflict in traits that are closely related to fitness.

### (d) Implications for studies on humans

Brown *et al*. [17] recently pointed out that although the majority of current evolutionary research on humans rests on the assumption of sex-specific benefits of multiple mating, few studies have directly addressed this, or actual differences between the sexes in selection on specific traits. In this study, the apparent sexual conflict at the phenotypic level was not reflected at the genetic level. The generally small sex differences in heritabilities and genetic correlations with fitness may reflect the monogamous mating system in preindustrial Finland. Because 87% of men and 90% of women in the analysed data were lifetime monogamous, an individual's reproduction was severely constrained by that of their partner. Thus, the timing and rate of reproduction are likely to result from a compromise between the two partners, leading to similar genetic correlations with fitness. Further, previous studies of preindustrial Finland have shown that while remarriage increased the number of children produced in men, it did not increase their number of grandchildren [58]. However, given the differential costs of reproduction between the sexes [21], the asymmetry in the benefits of maternal versus paternal care for offspring success [59], and the strong trade-off between offspring quality and quantity in humans [32], this should create opportunities for conflict at multiple levels, even in socially monogamous, biparental societies. Humans show a highly unusual level of diversity of mating systems across societies, ranging from strictly monogamous, to polygynous, polyandrous and polygynandrous [60] and there is large interpopulation variation in the ratio of male to female variance in reproductive success [17]. Populations also exhibit dramatic differences in the contributions of offspring quality versus quantity to parental fitness and the need for maternal versus paternal care for offspring success [59]. Thus, humans would seem to offer an unparalleled study system to investigate the influence and importance of the above factors for patterns of sexual conflict. A promising avenue would be to estimate the degree of sexual conflict on the genetic level in human populations that have a higher average number of lifetime partners (owing to, for example, polygyny or higher levels of extra-pair paternity). However, the great challenge for such endeavours lies in creating multigenerational pedigrees with known paternities.

## 5. Conclusion

Stearns *et al*. [22] recently stressed the importance of a genetic perspective in studies of selection and evolutionary change in humans. Our study illustrates how a genetic perspective can alter the interpretation of the degree of sexual conflict, because despite different selection pressures between the sexes on age at first and last reproduction and reproductive lifespan, there was no sexual conflict at the genetic level over these traits in this preindustrial human population. In general, while heritability estimates for diverse traits are accumulating rapidly in humans, more studies are needed that estimate genetic correlations between the sexes and between traits and fitness in both sexes.
